# Azocalix[4]arene-Rhodamine Supramolecular Hypoxia-Sensitive Systems: A Search for the Best Calixarene Hosts and Rhodamine Guests

**DOI:** 10.3390/molecules26185451

**Published:** 2021-09-07

**Authors:** Diana Mironova, Vladimir Burilov, Farida Galieva, Mohamed Ali Mohamed Khalifa, Sofia Kleshnina, Alsu Gazalieva, Ramil Nugmanov, Svetlana Solovieva, Igor Antipin

**Affiliations:** 1 Alexander Butlerov Institute of Chemistry, Kazan Federal University, 18 Kremlevskaya Street, 420008 Kazan, Russia; mir_din@mail.ru (D.M.); kleo-w@mail.ru (F.G.); chem.khalifa21@yahoo.com (M.A.M.K.); avaliyahmetova@mail.ru (A.G.); nougmanoff@hotmail.com (R.N.); iantipin54@yandex.ru (I.A.); 2 A.E.Arbuzov Institute of Organic & Physical Chemistry, 8 Arbuzov Street, 420088 Kazan, Russia; skleshni@iopc.ru (S.K.); evgersol@yandex.ru (S.S.)

**Keywords:** calixarene, hypoxia sensing, host-guest complex

## Abstract

A potential hypoxia-sensitive system host-guest complex of three calixarenes (including two with four anionic carboxyl and sulphonate azo fragments on the upper rim and a newly synthesized bis-azo adduct of calixarene in the cone configuration with azo fragments on the lower rim with the most widespread cationic and zwitterionic rhodamine dyes (123, 6G and B)) was studied using UV-VIS spectrometry and fluorescence as well as 1D and 2D NMR techniques. It was found that all three calixarenes form a complex with rhodamine dyes with a 1:1 composition. The association constants of calixarene-dye complexes with sulfonate calixarenes, especially in the case of tetra-anionic calixarene, turned out to be higher compared with carboxyl calixarene due to the more intense electrostatic interactions. For the first time using an HRESI MS technique, it was shown that the treatment of rhodamine 6G and 123 with sodium dithionite (SDT) produces a non-fluorescent leuco form of the dye, and only rhodamine B can be used with SDT without the occurrence of a side reduction. Moreover, it was identified that in addition to the reduction in the azo groups, SDT causes partial cleavage of the aryl ether bonds. The found features of SDT should be taken into account when SDT is used as an azoreductase mimic.

## 1. Introduction

**Scheme 1:**
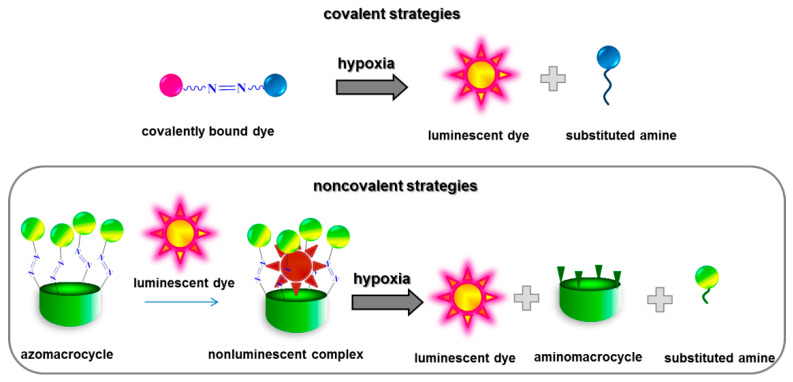
Two strategies for hypoxia-sensitive systems.

The main cellular energy processes that occur in the mitochondria depend on the normal concentration of oxygen in the tissues [1,2]. The phenomenon of hypoxia not only accompanies the development of many pathological conditions in the human body but is an important and characteristic manifestation of most large tumors, which is explained by an imbalance in oxygen consumption in normal and tumor cells due to changes in the tumor vasculature [3,4]. Hypoxia is an important indicator of aggressive tumors. Thus, a rapid visualization of hypoxia is one of the most important steps in the early diagnosis of various natural tumors. Moreover, systems that respond to hypoxia can be used not only for imaging but also for selective drug delivery and the effective treatment of tumors [5,6]. Usually, methods for hypoxia visualization are based on the “covalent” method, i.e., the formation of a covalent bond between an azo group compound and luminescence dye (Scheme 1) [7,8]. For this purpose, the initial azo compound should be non-luminescent, but in hypoxia conditions, a reduction in the azo group into an amino group releases luminescent dye, thus visualizing hypoxia [9]. To obtain non-luminescent dyes, conjugation with “black hole quenchers” is often used [10]. A selective reduction in nitroaromatic compounds by nitroreductase was also successfully used for hypoxia visualization [11,12].

Since the “covalent” method suffers from a complicated molecular design and time-consuming synthesis of the corresponding fluorescent probes, the “non-covalent” approach using host-guest chemistry is much easier (Scheme 1) [13,14]. In the past two years, the “non-covalent” approach using the host-guest complex of an anionic carboxylate calix[4]arene azo derivative and positively charged dye (rhodamine 123) was successfully used for hypoxia imaging in living cells [15]. Calixarene-embedded nanoparticles with the same hypoxia-responsive anionic carboxylate calix[4]arene and different molecular drugs in the cavity were successfully used for selective drug delivery and release in tumors [16]. However, the relationship of the structure of azo calixarenes and hypoxia response in such hypoxia-sensitive systems is still unclear. Moreover, an important question is the use of other fluorescent dyes, including more affordable ones. Herein, we expand the circle of hypoxia-sensitive systems, using three different azo-calix[4]arene derivatives and different report dyes. The association constant, the stoichiometry of host-guest complexes and their absorption and emission properties in terms of normoxia and hypoxia are discussed.

## 2. Results and Discussion

**Scheme 2:**
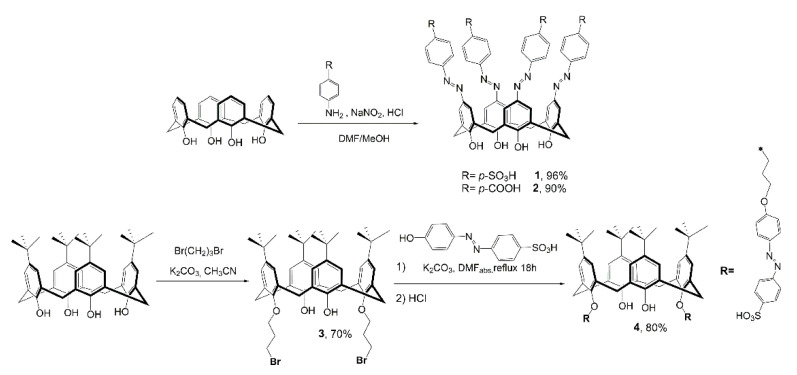
Synthetic pathway for macrocycles **1**, **2** and **4**.

The synthesis of azo-calix[4]arene derivatives bearing azobenzoic acid or azobenzenesulfonic acid fragments (compounds **1** and **2**, Scheme 2) was carried out using methods from the literature [17,18].

**Figure 1:**
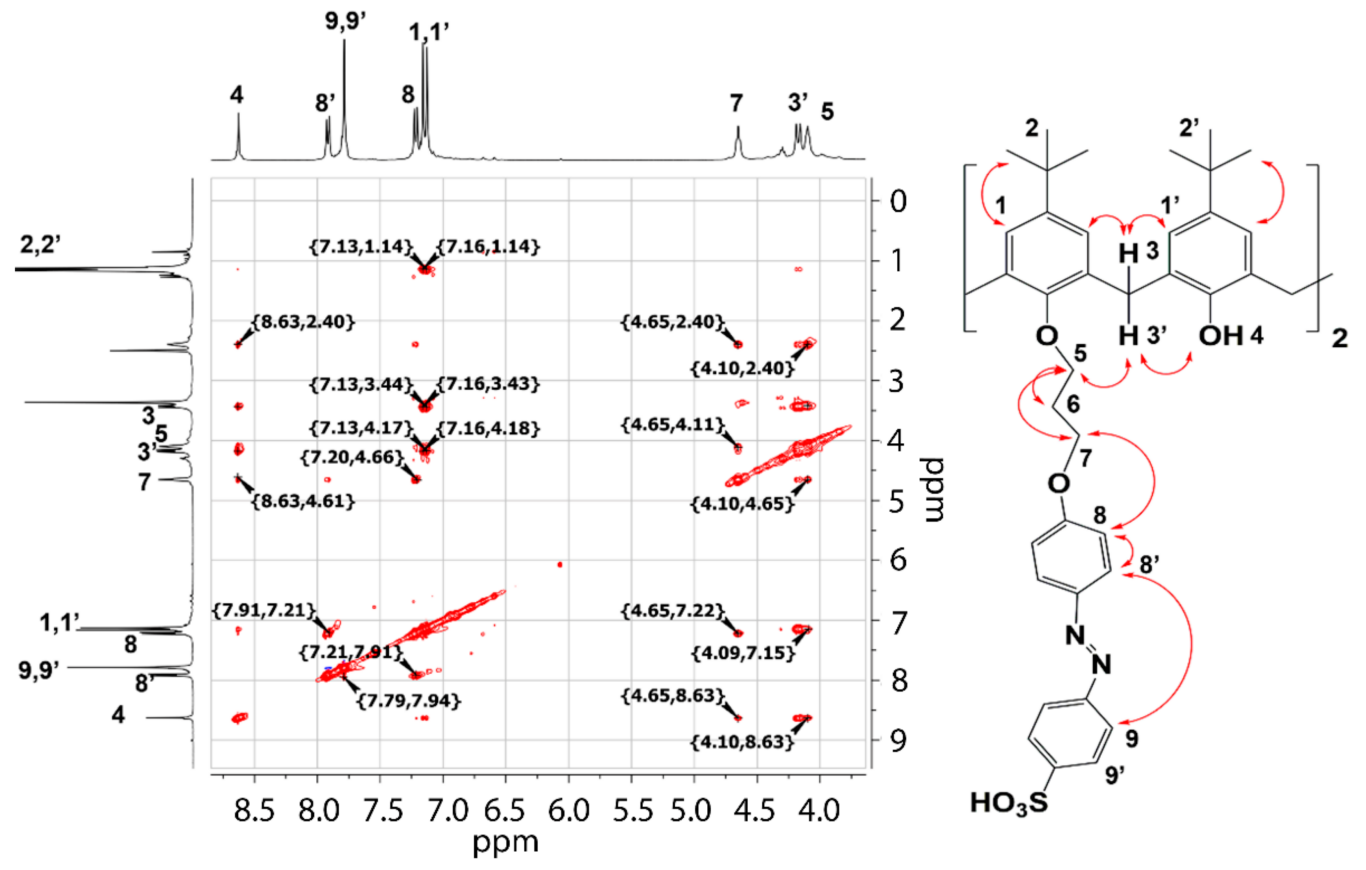
A fragment of 2D NOESY ^1^H-^1^H spectra of **4**, DMSO-d6, 25 °C.

To evaluate the role of azo fragments in the stability of host-guest calixarene-dye complexes, as well as the potential of their use for hypoxia imaging, macrocycle **4** containing two azo fragments was synthesized (Scheme 2). The synthesis of the initial bromopropyl calixarene derivative **3** as well as 4-[4-hydroxyphenylazo]-benzenesulfonic acid sodium salt was carried out using methods from the literature [19,20]. Compound **4** was obtained after refluxing the reaction mixture for 18 h with 80% yield. The structure of **4** was well established using 1D NMR ^1^H and ^13^C spectroscopy, as well as IR spectroscopy and high-resolution ESI mass spectrometry (HRESI). NMR signal assignment was performed using 2D NOESY ^1^H-^1^H spectroscopy. In the spectra (Figure 1), there are cross-peaks between the signals of aromatic protons 1 and 1′ with *tert*-butyl protons 2 and 2′ (δ = 7.13, 7.14 and 1.14 ppm), cross-peaks between the signals of axial methylene bridge proton 3 with aromatic protons (δ = 7.13, 7.14 and 3.44 ppm), cross-peaks between the signals of equatorial methylene bridge proton 3′ with propylene linker protons (δ = 4.16, and 4.65, 2.40 ppm) and cross-peaks between the signals of propylene linker protons with the aromatic protons of azo dye fragments (δ = 4.65, and 7.20, 7.91 ppm).

**Scheme 3:**
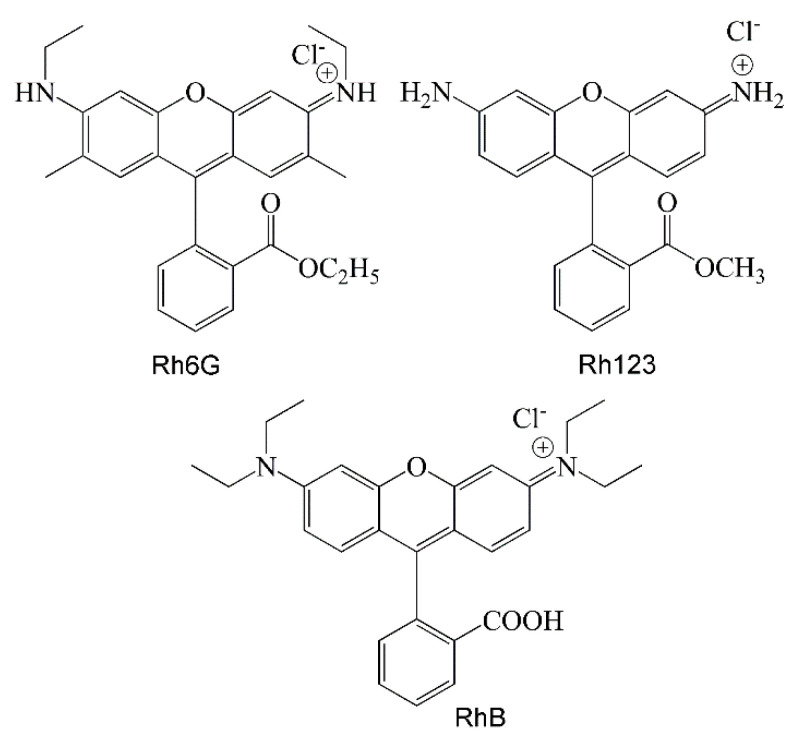
The structures of rhodamine dyes used in binary calixarene-dye systems.

In the HRESI mass spectra (Figure S1), in negative mode, the main peaks correspond to [M-2H]^2−^ with *m*/*z* = 641.2691 (calculated for C_74_H_82_N_4_O_12_S_2_^2−^ = 641.2691), [M-H]^1−^ with *m*/*z* = 1283.5449 (calculated for C_74_H_83_N_4_O_12_S_2_^−^ = 1283.5454) and [M-2H + Na]^1−^ with *m*/*z* = 1305.5307 (calculated for C_74_H_82_N_4_NaO_12_S_2_^2^ = 1305.5274). To create hypoxia-sensitive systems, the next step was the study of double azo calixarene-dye systems. Considering that all macrocycles contain negatively charged sulfonic or carboxylate groups, the most widespread xantene cationic dyes, namely rhodamines 6G, 123 and B, were used (Rh6G, Rh123 and RhB, Scheme 3).

**Figure 2:**
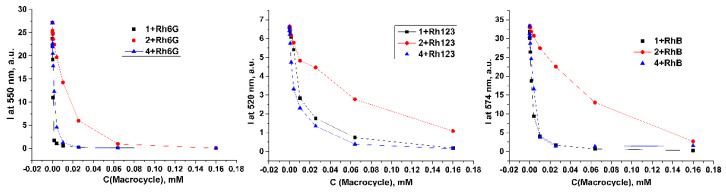
Fluorescent titration of Rh6G, Rh123 and RhB (1 μM) with macrocycles **1**, **2** and **4** in PBS buffer (pH 7.4) at 37 °C.

All of the used dye molecules exhibit a high brightness and photostability [21,22]. The fluorescence response of rhodamine dyes upon the addition of macrocycles **1**, **2** and **4** was determined according to fluorescence spectroscopy data (Figure 2). In all studied systems, the luminescence quenching of rhodamine dyes was observed after the addition of macrocycles.

**Figure 3:**
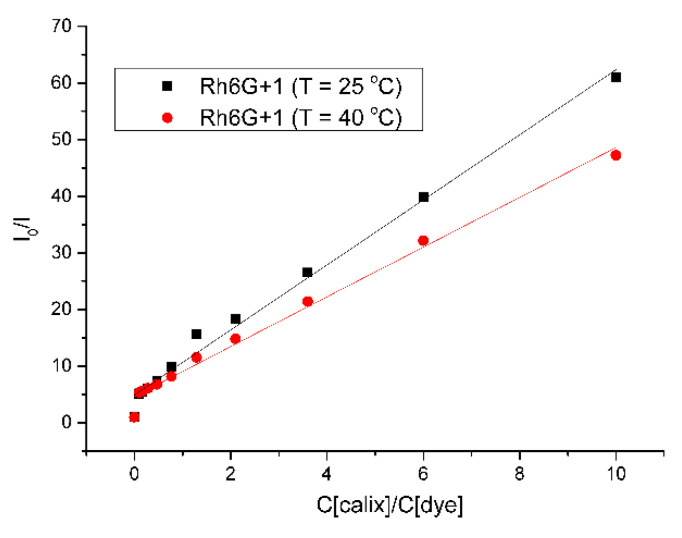
Stern-Volmer plot for Rh6G with macrocycle **1** at different temperatures. C (dye) = 1 μM, PBS buffer (pH 7.4).

In order to distinguish between the static and dynamic mechanisms of luminescence quenching, fluorescence titration at different temperatures was carried out. It is known [23] that in the case of static quenching, an increase in temperature facilitates the decomposition of the black complex, while in the case of dynamic quenching, an increase in temperature accelerates the frequency of particle collisions. According to the obtained data (Figure 3), an increase in temperature from 25 °C to 40 °C decreased the Rh6G fluorescence quenching in the binary Rh6G-**1** system, thus indicating static quenching as the main quenching mechanism and proving the formation of a calixarene-dye complex.

There are two general types of quenching mechanisms of fluorescence in the dye-calixarene complex—resonant Förster energy transfer (FRET) or photoinduced electron transfer (PET). The linearity of dependence of I_0_/I (I_0_—the emission intensity of the free dye, I—the emission intensity of the bound dye) on the concentration of the quencher using all macrocycles (Figure S2) indicates the prevalence of only one type of quenching mechanism. The FRET mechanism is excluded due to the absence of overlap of the absorption band of macrocycles with the emission band of the dye (Figure S3). Therefore, PET is the most likely quenching mechanism. To find the stoichiometry of the dye-macrocycle systems, Job’s plots were built (Figure S4). The binding stoichiometry for all systems was found to be 1:1. Thus, it is obvious that electrostatic interactions are the driving force of the formation of such complexes. However, the structure of the dye and the nature of azo fragments on the calixarene platform also play an important role. To compare azo calixarene and rhodamine dyes between each other, the association constant (K_a_) was determined by fitting the data of the fluorescence titration (Table 1).

The association constants of calixarenes containing sulfonate fragments turned out to be higher in the interaction with all dyes compared to macrocycle **2** with carboxyl ones. Even macrocycle **4**, which has only two sulfonate residues, turned out to be more efficient in binding with rhodamine dyes than the tetracarboxy-substituted macrocycle **2**. Previously, it was shown that the distribution of the electrical charge over sulfate as well as sulfonate groups is rather different from carboxylate headgroups, with the charge of the sulfate/sulfonate headgroup being larger than that of the carboxylate one [24,25]. Moreover, the pyramidal structure of the sulfonate anion results in a more efficient interaction with ammonium cations [26]. Despite the apparently higher charge in the alkylsulfonate and alkylsulfate derivatives, there is lack of comparative charge distribution in arylsulfonates and arylcarboxylates in the literature. We calculated APT (atomic polar tensor) and Mulliken charges using density functional theory (DFT)-based calculations with a ωB97XD functional, 6-311+g(d,p) basis set and SMD solvation model (water) (Figure S5). The total negative charge on oxygen atoms was observed to be −4.425 and −3.055 (Mulliken) or −1.372 and −1.250 for thequarter model of calixarenes **1** and **2**, respectively. Thus, the sulfonate aromatic derivatives are capable of more intense electrostatic interactions compared with the carboxylate ones.

**Figure 4:**
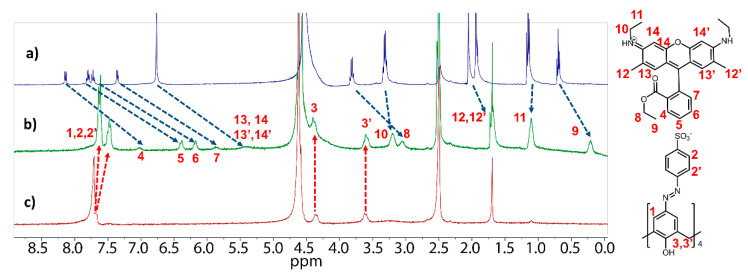
^1^H NMR spectra of 0.5 mM Rh6G (**a**), 1 mM calixarene **1** (**c**) and their (0.5:1) mixture (**b**) in DMSO-d6:D_2_O (2:3) at 25 °C, 10 mM PBS buffer (pD 7.4).

The ability of macrocycle **1** to form complexes with Rh6G was proved by ^1^H NMR spectroscopy. In the presence of macrocycle 1, Rh6G protons showed field shifts (Figure 4) caused by the ring current effect of the aromatic nuclei of the macrocycles.

The largest shifts are observed for the aromatic fragment of the Rh6G dye carrying the ester fragment (protons 4, 5, 6 and 7 have ∆δ = 1.09, 1.51, 1.55 and 1.48 ppm, respectively) as well as for the ethoxy group protons (8 and 9 have ∆δ = 0.77 and 0.50 ppm, respectively). It is noteworthy that the protons of the xanthene fragment also undergo a significant upfield shift (protons 13 and 14 as well as 12 have ∆δ = 1.73 and 0.38 ppm, respectively), while the proton signals of the aminoethyl fragment are less shifted (protons 10 and 11 have ∆δ = 0.11 and 0 ppm, respectively). Thus, it becomes obvious that the dye is immersed into the cavity of the macrocycle with its benzene moiety, forming a guest–host complex, while the positively charged xanthene moiety is located outside. It should be noted that the values of the high-field shifts of the dye proton signals are significantly greater than those for the **2**-Rh123 system, previously studied in the Guo group [15], which is consistent with the stability constants of the complexes we obtained and indicates a stronger interaction of sulfonate calixarene with the rhodamine dyes. The interaction of macrocycle 1 with rhodamine was also confirmed by the method of 2D NOESY ^1^H-^1^H spectroscopy (Figure S6). The presence of cross-peaks between the aromatic protons of **1** (δ = 7.60 and 7.41 ppm) with the protons of the benzene moiety of Rh6G (δ = 6.40 and 6.17 ppm) as well as with Rh6G ethoxy protons (δ = 3.53 and 0.23 ppm) unambiguously proves the formation of a guest-host complex. According to the ^1^H NMR data (Figure S7), in the case of the calixarene **1**-RhB system, the interaction occurs predominantly with the xanthene part of the dye: for example, the protons of the xanthene part upshift to 0.18 ppm followed by the upfield shifts of ethylamine fragments. Compared to the upfield shifts of Rh6G, it can be concluded that the interaction with RhB is much less efficient than that for Rh6G.

**Figure 5:**
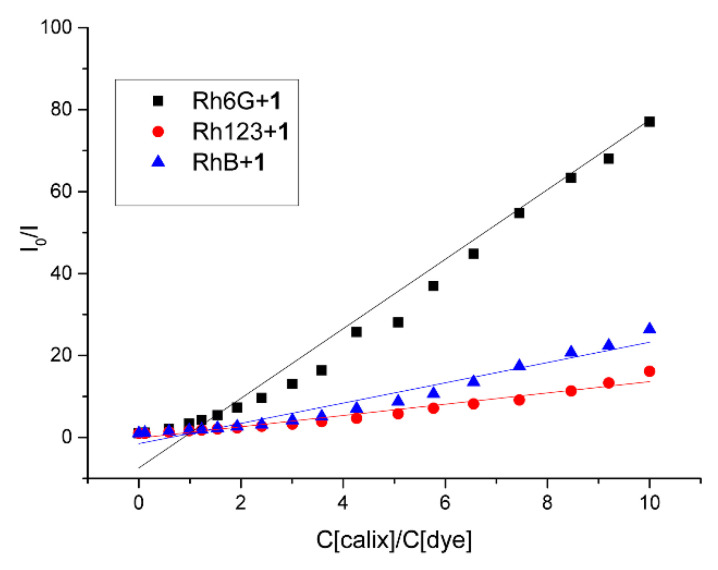
Stern-Volmer plots for rhodamine dyes with macrocycle **1** at pH 4.5. C (dye) = 1 μM, acetate buffer (pH 4.5).

The difference in the interaction of dyes with macrocycles turned out to be quite unusual. Thus, K_a_ for the complexes of all studied calixarenes with Rh6G was found to be higher than that for Rh123 and RhB, especially in the complex with calixarene **1** (K = 1.45 × 10^7^ with Rh6G instead of 5.03 × 10^5^ with Rh123). Taking into account the low pK_a_ value of RhB (pK_a_ = 3.7 [27]), it becomes clear that RhB at pH 7.4 is in the neutral zwitterionic form, which causes less effective electrostatic interactions and, thus, explains our NMR data: the RhB molecule interacts with macrocycle **1** with the more bulky xanthene part, while the negatively charged benzene fragment carrying the carboxylate group is turned outward from the macrocycle cavity. However, the pK_a_ values of Rh123 and Rh6G are close enough (pK_a_ = 6.1 [28] and 7.5 [29], respectively) to be presented in the cationic form at pH 7.4. Nevertheless, a fluorescence titration at pH 4.5 was performed to remove the factor of pK_a_ difference between Rh123 and Rh6G (Figure 5). The general trend turned out to be the same as at pH 7.4: Rh6G was found to be the most effective binder. In numerical terms, however, the constant with Rh123 and RhB increased by one order of magnitude, while the constant with Rh6G had a twofold decrease (Table 1 and Table 2).

Thus, the preferred interaction of macrocycles **1**, **2** and **4** with Rh6G cannot be explained only from the standpoint of electrostatic interactions. Taking into account the great difference in the logP values of Rh6G and Rh123 (6.5 and 1.5), as well as the difference in logD values (2.1 and 0.5, respectively) [30], it can be assumed that the comparatively high hydrophobicity of Rh6G favors the interactions with the hydrophobic basket of calixarene.

**Figure 6:**
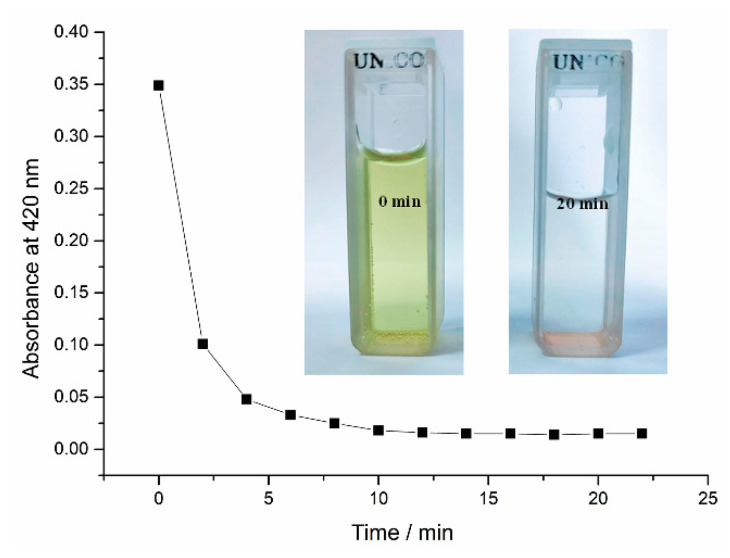
Absorbance at 420 nm of macrocycle **1** (10 μM) as a function of time following the addition of SDT (1.0 mM), PBS buffer, pH 7.4, 37 °C.

To test binary calixarene-dye systems for the hypoxia process, UV/Vis spectroscopy was employed. All three macrocycles have a broad absorption peak in the 400–500 nm range (Figure S8), attributed to the n-π* transitions of the azo groups. In the case of the addition of excess of sodium dithionite (SDT), which acts as a chemical mimic of azoreductase [31,32], a loss of the characteristic yellow color of the macrocycle was detected within 7 min (photos in Figure 6).

The instant disappearance of the azo absorption indicates a complete reduction reaction, thus indicting a reduction in all the azo groups of the macrocycles. The kinetics of reducing the azo group for macrocycles **1**, **2** and **4** through the absorbance at 420 nm in real time (Figure 6) were established. The attenuation curves of the intensity were successfully described by the quasi-first-order reaction decay model. In Table 3, the calculated constant values for the azo group reduction are presented. The reduction constants for tetrasubstituted macrocycles **1** and **2** are lower than those for disubstituted macrocycle **4**, which might be related to the reduced amount of azo groups in the case of calixarene **4**.

**Figure 7:**
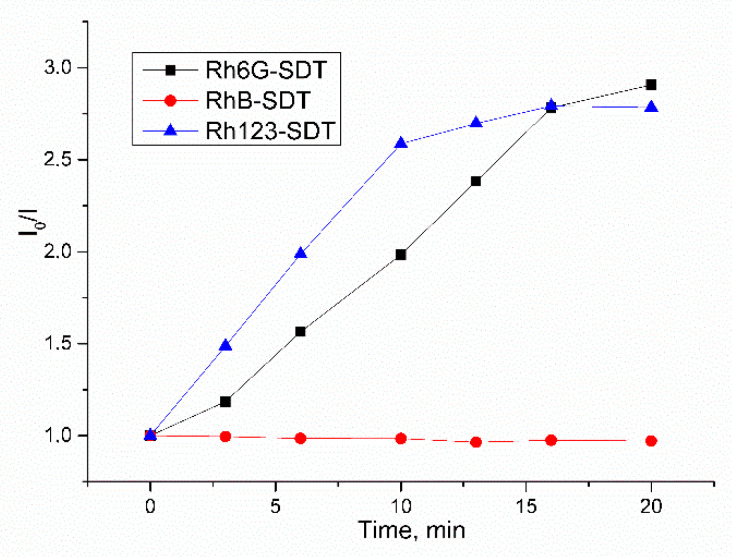
I_0_/I vs. time for rhodamine dyes in the presence of SDT as a function of time. C (dye) = 1 μM, C (SDT) = 100 μM, PBS buffer (pH 7.4).

Before the transition to ternary systems, we studied the stability of the selected rhodamine dyes to the SDT. Surprisingly, after 20 min of incubation with a 100-fold excess of SDT, the fluorescence of Rh123 and Rh6G was quenched threefold (Figure 7).

Considering that Rh123 has already been used in the literature in combination with SDT [15], this result was unexpected and required additional experiments. After treating the solutions with SDT, they were analyzed using HRESI mass spectrometry combined with liquid chromatography. In the case of Rh123, analyzed in positive mode, we found two components: the first one was found to be Rh123, having [M + H]^+^ with *m*/*z* = 345.1241 (calculated for C_21_H_17_N_2_O_3_^+^ = 345.1234), and another compound had *m*/*z* = 345.1392 (calculated for C_21_H_19_N_2_O_3_^+^ = 347.1391) and *m*/*z* = 369.1211 (calculated for C_21_H_18_N_2_NaO_3_^+^ = 369.1215), which corresponds to the leuco form of Rh123, reduced by SDT (Figure S9). In the case of Rh6G, we also found the initial dye and its leuco form ([M + H]^+^ with *m*/*z* = 443.2336 (calculated for C_28_H_31_N_2_O_3_^+^ = 443.2330) for Rh6G and [M + H]^+^ with *m*/*z* = 443.2487 (calculated for C_28_H_33_N_2_O_3_^+^ = 443.2487) for its leuco form, respectively) (Figure S10). In the case of RhB, no formation of a leuco form upon treatment with SDT was observed. Thus, for the first time, we discovered the formation of a non-luminescent leuco form of Rh123 and Rh6G under treatment with SDT, which, nevertheless, requires a separate study, since it is not clear whether dithionite itself or its decomposition products act as reduction agents.

**Figure 8:**
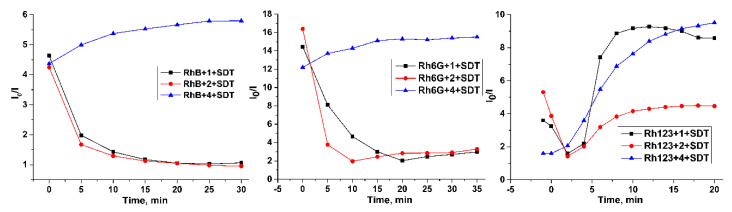
I_0_/I vs. time for rhodamine dye-calixarene systems in the presence of SDT. C (dye) = 1 μM, C (**1**, **2**, **4**) = 1 μM, C (SDT) = 100 μM, PBS buffer (pH 7.4).

Thus, during the transition to double calixarene-dye systems (Figure 8), it became expected that the interaction of the rhodamine dye itself with SDT would make a significant contribution, leading to the quenching of the luminescence of the released dye. Indeed, in the case of double Rh123-**1** and Rh123-**2** systems at the fifth minute, we observed a full release of the dye with the restoration of its intensity to the level of the free form (when I_0_/I = 1). However, after 10 min, a 10-fold luminescence quenching of Rh123 was observed, which can be attributed to the decomposition of the released dye to its leuco form. In the case of Rh6G, the decomposition of the dye is not so pronounced but also takes place after 20 min. It is noteworthy that in the case of Rh123, the quenching of luminescence in the presence of calixarenes is significantly higher, which may indicate the mediator role of calixarene in the redox reaction with the dye. Moreover, only in the case of RhB is there a complete release of the dye, followed by an increase in emission to the level of the free dye. Macrocycle **4** after exposure with SDT did not release the rhodamine dyes. This can only be the case if the reduced form of the macrocycle proceeds to bind the dye. To study the interaction of the dye with the reduced form of macrocycle **4**, it was treated with an excess of sodium dithionite and isolated and analyzed using HRESI mass spectrometry combined with liquid chromatography (Figure S11).

**Scheme 4:**
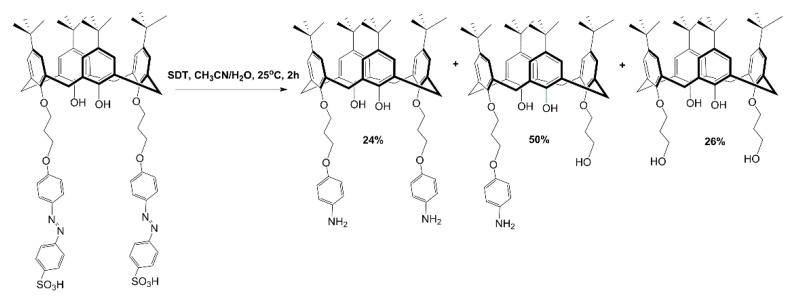
Decomposition of 4 upon treatment with 100-fold excess of SDT.

In addition to the desired product of the azo bond cleavage with *m*/*z* = 969.5751, which corresponds to the [M + Na]^+^ (calculated for C_62_H_78_N_2_NaO_6_^+^ = 969.5758), we also found two products of the ether bond cleavage: a mono-cleavage product with *m*/*z* = 878.5334, which corresponds to the [M-C_6_H_5_N + Na]^+^ (calculated for C_56_H_73_NNaO_6_^+^ = 878.5336), and a di-cleavage product with *m*/*z* = 787.4916, which corresponds to the [M-C_12_H_10_N_2_ + Na]^+^ (calculated for C_50_H_68_NaO_6_^+^ = 787.4908) (Scheme 4). Thus, it becomes obvious that SDT is capable of cleaving the aryl ether bond, which was also recently shown by Cybulska [33] in the reductive cleavage of β-O-4 bonds in lignin with the consequent formation of phenolic monomers. Indeed, the addition of an equimolar amount of a mixture of the reduction products of calixarene 4 caused a fivefold quenching of the luminescence for all three rhodamine dyes. Thus, the reduction products of calixarene 4 are capable of re-binding the released dye, making the usage of such structures impossible for hypoxia sensing. However, it is still unclear exactly which reduction products bind with the rhodamine dyes. Therefore, the features of the cleavage of the aryl ether bond on the calixarene platform by SDT as well as the interaction features of the cleavage products with the rhodamine dyes require a separate study, which is currently in progress.

## 3. Materials and Methods

### 3.1. Sample Preparation

The phosphate-buffered saline (PBS) solution with pH 7.4 was prepared by dissolving powder (Sigma P-3813) in 1000 mL of Milli-Q water. The stock solutions (100 μM) of macrocycles and dyes were prepared by dissolving the corresponding chemicals in PBS buffer. The fluorescence titrations were performed by a successive addition of known amounts of macrocycles to the dye solution in quartz cuvette. Argon gas was bubbled into the solution for 30 min to create the hypoxic environment.

### 3.2. Apparatus

The NMR spectra were recorded on Bruker Avance 400 Nanobay (Bruker Corporation, Billerica, MA, USA) with signals from residual protons of deuterated solvents (CDCl_3_ or DMSO-d6) as the internal standard. The UV-Vis spectra were recorded in a quartz cell (light path 10 mm) on a Shimadzu UV-2600 UV-Vis spectrophotometer (Shimadzu Corporation, Kyoto, Japan) equipped with a Cary dual-cell Peltier accessory. The fluorescence measurements were recorded in a conventional quartz cell (light path 10 mm) on a Fluorolog FL-221 spectrofluorimeter (Horiba, Ltd, Kyoto, Japan) equipped with a single-cell Peltier accessory. The HRESI experiments were performed with an Agilent 6550 iFunnel Q-TOF LC/MS (Agilent Technologies, Santa Clara, CA, USA), equipped with Agilent 1290 Infinity II LC. Analytical chromatography was performed on a ZORBAX Eclipse plus C18 column 2.1*50 mm 1.8 micron (Agilent Technologies, Santa Clara, CA, USA) using H_2_O-MeOH (20:80) or CH_3_CN-MeOH (80:20) as the eluent. The IR spectra were recorded on a Bruker Vector-22 spectrometer (Bruker Corporation, Billerica, MA, USA). The samples were prepared by suspending them in mineral oil or as thin films, obtained from chloroform solutions dried on the surface of the KBr tablet. The melting points were measured using the Stuart SMP10 (Stuart Scientific, Stone, UK).

### 3.3. Quantum Chemical Calculations

The quantum chemical calculations were performed in the Gaussian-09 [34] package. The DFT level of theory with a ωB97XD functional, 6-311+g(d,p) basis set and SMD solvation model (water) was used. Structure conformations were enumerated in rdkit [35] and optimized to minima. The lowest energy conformers were chosen for analysis.

### 3.4. Synthesis

All reagents and solvents were purchased from either Acros (Geel, Belgium) or Sigma-Aldrich (Burlington, VT, USA) and used without further purification. 5,11,17,23-tetrakis[(4-sulfophenyl)azo]-25,26,27,28-tetrahydrocalix[4]arene **1** [17] and 5,11,17,23-tetrakis[(4-carboxyphenyl)azo]-25,26,27,28-tetrahydrocalix[4]arene **2** [18] as well as 25,27-bis(3-bromopropoxy)-26,28-dihydroxy-5,11,17,23-tetra(tert-butyl)calix[4]arene **3** [19] and 4-[4-hydroxyphenylazo]-benzenesulfonic acid sodium salt [20] were synthesized according to published methods.

25,27-bis(3-(4-((4-sulfophenyl)diazenyl)phenoxy)propyl)-26,28-dihydroxy-5,11,17,23-tetra(tert-butyl)calix[4]arene **4**

Compound **3** (0.3 mmol) was suspended in DMF (15 mL), and then 4-[4-hydroxyphenylazo]-benzenesulfonic acid sodium salt (1 mmol) and K_2_CO_3_ (1 mmol) were added. The mixture was refluxed for 18 h under an inert atmosphere. Then, the reaction mixture was evaporated; the following residue was treated with HCl_(conc.)_; and the forming precipitate was filtered, washed with H_2_O (3 × 20 mL) and dried in vacuo to give compound **4** (0.34 g, 80%) as a dark orange powder.

mp 240 °C (dec.). R_f_ (acetic acid:hexane = 1.5:1) 0.45.

IR (KBr) ν_max_ cm^−1^: 3417 (OH), 1485 (N=N), 1362 (S=O).

^1^H NMR (400 MHz, DMSO-*d*_6_, 25 °C) δ_H_ ppm: 8.63 (s, 2H, OH), 7.92 (d, *J* = 8.8 Hz, 4H, ArH), 7.79 (m, 8H, ArH), 7.21 (d, *J* = 8.8 Hz, 4H), 7.16 (s, 4H, ArH), 7.13 (s, 4H, ArH), 4.65 (t, *J* = 5.7 Hz, 4H, CH_2_O), 4.16 (d, *J* = 12.7 Hz, 4H, CH_2_), 4.12 (t, *J* = 5.7 Hz, CH_2_O), 3.43 (d, *J* = 12.7 Hz, 4H, CH_2_), 2.40 (m, 4H, CH2), 1.16 (s, 18H, *t*-Bu), 1.13 (s, 18H, *t*-Bu).

^13^C NMR: (100.6 MHz, DMSO-*d*_6_, 25 °C) δ_c_ ppm: 161.9, 152.21, 150.75, 150.59, 149.92, 147.75,146.73, 141.91, 133.71, 127.90, 127.14, 126.20, 125.81, 125.21, 122.23, 115.58, 73.11, 65.14, 34.52, 34.07, 31.87, 31.69, 31.39, 31.25.

HRESI MS: calculated for [M-2H]^2−^ C_74_H_82_N_4_O_12_S_2_^2−^ = 641.2691, found *m*/*z* = 641.2691, calculated for [M-H]^1−^ C_74_H_83_N_4_O_12_S_2_^−^ = 1283.5454 found *m*/*z* = 1283.5449, calculated for [M-2H + Na]^1−^ C_74_H_82_N_4_NaO_12_S_2_^2^ = 1305.5274 found *m*/*z* = 1305.5307.

## 4. Conclusions

In addition to the known anionic tetra-azo calixarenes with azo fragments on the upper rim, a bis-azo adduct of calixarene in the cone configuration with azo fragments on the lower rim was synthesized for the first time. A systematic study of calixarene-rhodamine binary systems using UV-VIS spectrometry and fluorescence, as well as an NMR technique, was carried out. It was found that in all cases, dye-calixarene complexes with a 1:1 composition were formed. The association constants of calixarene-dye complexes with sulfonate calixarenes turned out to be higher compared with carboxyl ones. Using DFT calculations, it was shown that the sulfonate aromatic derivatives are capable of more intense electrostatic interactions compared with the carboxylate ones. Among rhodamine 6G, B and 123, it was found that the comparatively high hydrophobicity of rhodamine 6G favors the interactions with the hydrophobic basket of calixarene. For the first time using an HRESI MS technique, it was shown that the treatment of rhodamine 6G and 123 with SDT produces a non-fluorescent leuco form of the dye. Moreover, it was shown that in addition to the azo group reduction, SDT causes partial cleavage of the aryl ether bonds, which should be taken into account when SDT is used as an azoreductase mimic.

## Figures and Tables

**Table 1:** ^a^ Association constants (K_a_) of calixarene **1**, **2** and **4** complexes with rhodamine dyes.

System	K_a_, M^−1^
**1**-Rh123	5.0 ± 0.3 × 10^5^
**2**-Rh123	2.3 ± 0.6 × 10^4^
**4**-Rh123	2.4 ± 0.5 × 10^5^
**1**-Rh6G	1.5 ± 0.1 × 10^7^
**2**-Rh6G	1.0 ± 0.4 × 10^5^
**4**-Rh6G	1.0 ± 0.1 × 10^6^
**1**-RhB	7.0 ± 0.1 × 10^5^
**2**-RhB	2.6 ± 0.1 × 10^4^
**4**-RhB	3.6 ± 0.4 × 10^5^

^a^ C (dye) 1 μM, (macrocycle) 0–0.16 mM, PBS buffer (pH 7.4), 37 °C.

**Table 2:** ^a^ Association constants (K_a_) of calixarene 1 complexes with rhodamine dyes at pH 4.5.

System	K_a_, M^−1^
**1**-Rh123	1.1 ± 0.1 × 10^6^
**1**-Rh6G	6.8 ± 0.7 × 10^6^
**1**-RhB	1.5 ± 0.1 × 10^6^

^a^ C (dye) 1 μM, (macrocycle) 0–0.16 mM, acetate buffer (pH 4.5), 37 °C.

**Table 3:** Kinetics of the reduction in macrocycles **1**, **2** and **4** in the presence of SDT.

Macrocycle	*k*, min^−1^
**1**	0.350 (Adj. R^2^ > 0.991)
**2**	0.403 (Adj. R^2^ > 0.987)
**4**	0.614 (Adj. R^2^ > 0.982)

## Supplementary Materials

The following are available online. **Figure S1**: NMR ^1^H (a), ^13^C (b), IR (c) and HRESI-MS (c) spectra of 25,27-bis(4-((4-sulfophenyl)diazenyl)phenyl)-propoxy)-26,28-dihydroxy-5,11,17,23-tetra(tert-butyl)calix[4]arene **4**; **Figure S2**: Stern Volmer plots for Rhodamine 6G with macrocycles **1**, **2**, **4**. C (dye) = 1 μM, PBS buffer (pH 7.4) at 37 °C; **Figure S3**: Normalized emission spectrum of Rho6G and absorption spectrum of **1** in PBS buffer (pH 7.4) at 37 °C; **Figure S4**: Job’s plot for Rhodamine dyes with macrocycle **1**. C [total] = 2 μM, PBS buffer (pH 7.4) at 37 °C; **Figure S5**: Mulliken and APT charges in model 4-((4-hydroxyphenyl)diazenyl)benzoate (left) and 4-((4-hydroxyphenyl)diazenyl)benzenesulfonate (right); **Figure S6**: A fragment of 2D NOESY ^1^H-^1^H spectra of **1** with Rh6G, 0.5 mM Rho6G and 1 mM of calixarene in DMSO-d6:D_2_O (2:3) at 25 °C, 10 mM PBS buffer (pH 7.4); **Figure S7**: ^1^H NMR spectra of 0.5 mM RhoB (a), 1 mM calixarene **1** (c) and their (0.5:1) mixture (b) in DMSO-d6:D_2_O (2:3) at 25 °C, 10 mM PBS buffer (pH 7.4); **Figure S8**: Absorption spectra of macrocycles **1**, **2**, **4** in PBS buffer; **Figure S9**: Chromatogram and HRESI-mass spectra of Rh123, treated with 100-fold excess of SDT; **Figure S10**: Chromatogram and HRESI-mass spectra of Rh6G, treated with 100-fold excess of SDT; **Figure S11**: Chromatogram and HRESI-mass spectra of **4**, treated with 100-fold excess of SDT.

## Sample Availability

Samples of the compounds **1**, **2** and **4** are available from the authors.

## References

[1] Wilson W.R., Hay M.P. (2011). Targeting hypoxia in cancer therapy. Nat. Rev. Cancer.

[2] Gatenby R.A., Gillies R.J. (2008). A microenvironmental model of carcinogenesis. Nat. Rev. Cancer.

[3] Busk M., Overgaard J., Horsman M.R. (2020). Imaging of Tumor Hypoxia for Radiotherapy: Current Status and Future Directions. Semin. Nucl. Med..

[4] Harris A.L. (2002). Hypoxia—a key regulatory factor in tumour growth. Nat. Rev. Cancer.

[5] Zhang T.-X., Zhang Z.-Z., Yue Y.-X., Hu X.-Y., Huang F., Shi L., Liu Y., Guo D.-S. (2020). A General Hypoxia-Responsive Molecular Container for Tumor-Targeted Therapy. Adv. Mater..

[6] Pan Y.-C., Hu X.-Y., Guo D.-S. (2020). Biomedical Applications of Calixarenes: State of the Art and Perspectives. Angew. Chem. Int. Ed..

[7] Chevalier A., Piao W., Hanaoka K., Nagano T., Renard P.-Y., Romieu A. (2015). Azobenzene-caged sulforhodamine dyes: A novel class of ‘turn-on’ reactive probes for hypoxic tumor cell imaging. Methods Appl. Fluores..

[8] Sun L., Li G., Chen X., Chen Y., Jin C., Ji L., Chao H. (2015). Azo-Based Iridium(III) Complexes as Multicolor Phosphorescent Probes to Detect Hypoxia in 3D Multicellular Tumor Spheroids. Sci. Rep..

[9] Piao W., Tsuda S., Tanaka Y., Maeda S., Liu F., Takahashi S., Kushida Y., Komatsu T., Ueno T., Terai T. (2013). Development of Azo-Based Fluorescent Probes to Detect Different Levels of Hypoxia. Angew. Chem. Int. Ed..

[10] Uddin M.I., Evans S.M., Craft J.R., Marnett L.J., Uddin M.J., Jayagopal A. (2015). Applications of Azo-Based Probes for Imaging Retinal Hypoxia. ACS Med. Chem. Lett..

[11] Chen S., Liu J., Li Y., Wu X., Yuan Q., Yang R., Zheng J. (2020). Hypoxia-responsive fluorescent nanoprobe for imaging and cancer therapy. TrAC Trends Anal. Chem..

[12] Kim H.S., Sharma A., Ren W.X., Han J., Kim J.S. (2018). COX-2 Inhibition mediated anti-angiogenic activatable prodrug potentiates cancer therapy in preclinical models. Biomaterials.

[13] Yu J., Qi D., Li J. (2020). Design, synthesis and applications of responsive macrocycles. Commun. Chem..

[14] Wang Y.-Y., Kong Y., Zheng Z., Geng W.-C., Zhao Z.-Y., Sun H., Guo D.-S. (2019). Complexation of a guanidinium-modified calixarene with diverse dyes and investigation of the corresponding photophysical response. Beilstein J. Org. Chem..

[15] Geng W.-C., Jia S., Zheng Z., Li Z., Ding D., Guo D.-S. (2019). A Noncovalent Fluorescence Turn-on Strategy for Hypoxia Imaging. Angew. Chem. Int. Ed..

[16] Liu Q., Zhang T.-X., Zheng Y., Wang C., Kang Z., Zhao Y., Chai J., Li H.-B., Guo D.-S., Liu Y. (2021). Calixarene-Embedded Nanoparticles for Interference-Free Gene–Drug Combination Cancer Therapy. Small.

[17] Morita Y., Agawa T., Kai Y., Kanehisa N., Kasai N., Nomura E., Taniguchi H. (1989). Syntheses and Crystal Structure of Calix[4]quinone. Chem. Lett..

[18] Menon S.K., Patel R.V., Panchal J.G. (2009). Self-Aggregation and Solubilizing Properties of the Supramolecular System Based on Azobenzenesulfonate Calix[4]arene and CTAB. J. Incl. Phenom. Macrocycl. Chem..

[19] Li Z.-T., Ji G.-Z., Zhao C.-X., Yuan S.-D., Ding H., Huang C., Du A.-L., Wei M. (1999). Self-Assembling Calix[4]arene [2]Catenanes. Preorganization, Conformation, Selectivity, and Efficiency. J. Org. Chem..

[20] Wei Y., Zeng Q., Bai S., Wang M., Wang L. (2017). Nanosized Difunctional Photo Responsive Magnetic Imprinting Polymer for Electrochemically Monitored Light-Driven Paracetamol Extraction. ACS Appl. Mater. Interfaces..

[21] Rajasekar M. (2021). Recent Trends in Rhodamine derivatives as fluorescent probes for biomaterial applications. J. Mol. Struct..

[22] Gill H., Gokel M.R., McKeever M., Negin S., Patel M.B., Yin S., Gokel G.W. (2020). Supramolecular pore formation as an antimicrobial strategy. Coord. Chem. Rev..

[23] Lakowicz J.R. (2006). Principles of Fluorescence Spectroscopy.

[24] Zana R., Schmidt J., Talmon Y. (2005). Tetrabutylammonium Alkyl Carboxylate Surfactants in Aqueous Solution:  Self-Association Behavior, Solution Nanostructure, and Comparison with Tetrabutylammonium Alkyl Sulfate Surfactants. Langmuir.

[25] Reshetnyak E.A., Chernysheva O.S., Nikitina N.A., Loginova L.P., Mchedlov-Petrosyan N.O. (2014). Activity coefficients of alkyl sulfate and alkylsulfonate ions in aqueous and water-salt premicellar solutions. Colloid J..

[26] Burilov V.A., Mironova D.A., Ibragimova R.R., Nugmanov R.I., Solovieva S.E., Antipin I.S. (2017). Detection of sulfate surface-active substances via fluorescent response using new amphiphilic thiacalix[4]arenes bearing cationic headgroups with Eosin Y dye. Colloids Surf. A Physicochem. Eng. Aspects.

[27] Fang Y., Zhou A., Yang W., Araya T., Huang Y., Zhao P., Johnson D., Wang J., Ren Z.J. (2018). Complex Formation via Hydrogen bonding between Rhodamine B and Montmorillonite in Aqueous Solution. Sci. Rep..

[28] Wang P., Cheng M., Zhang Z. (2014). On different photodecomposition behaviors of rhodamine B on laponite and montmorillonite clay under visible light irradiation. J. Saudi Chem. Soc..

[29] Li M., Harbron R.L., Weaver J.V.M., Binks B.P., Mann S. (2013). Electrostatically gated membrane permeability in inorganic protocells. Nature Chem..

[30] Kennedy A.R., Conway L.K., Kirkhouse J.B.A., McCarney K.M., Puissegur O., Staunton E., Teat S.J., Warren J.E. (2020). Monosulfonated Azo Dyes: A Crystallographic Study of the Molecular Structures of the Free Acid, Anionic and Dianionic Forms. Crystals.

[31] Jaffe C.L., Lis H., Sharon N. (1980). New cleavable photoreactive heterobifunctional cross linking reagents for studying membrane organization. Biochemistry.

[32] Leriche G., Budin G., Brino L., Wagner A. (2010). Optimization of the Azobenzene Scaffold for Reductive Cleavage by Dithionite; Development of an Azobenzene Cleavable Linker for Proteomic Applications. Eur. J. Org. Chem..

[33] Brienza F., Van Aelst K., Thielemans K., Sels B.F., Debecker D.P., Cybulska I. (2021). Enhancing lignin depolymerization via a dithionite-assisted organosolv fractionation of birch sawdust. Green Chem..

[34] Frisch M.J., Trucks G.W., Schlegel H.B., Scuseria G.E., Robb M.A., Cheeseman J.R., Scalmani G., Barone V., Mennucci B., Petersson G.A. (2013). Gaussian 09, Revision D.01.

[35] Rdkit. https://www.rdkit.org/.

