# Supplementary material for: Azocalix[4]arene-Rhodamine Supramolecular Hypoxia-Sensitive Systems: A Search for the Best Calixarene Hosts and Rhodamine Guests
Source: Molecules. 2021 Sep 7;26(18):5451. doi: 10.3390/molecules26185451 (PMC8469542; doi:10.3390/molecules26185451)
Supplement: Supplementary file 1 [file molecules-26-05451-s001.zip › molecules-1363842-supplementary.pdf]

molecules

Supplementary information

Azocalix[4]arene-Rhodamine Supramolecular Hypoxia-Sensitive Systems: A Search for the Best Calixarene Hosts and Rhodamine Guests

Diana Mironova [1], Vladimir Burilov [1,*], Farida Galieva [1], Mohamed Ali Mohamed Khalifa [1], Sofia Kleshnina [2], Alsu Gazalieva [1], Ramil Nugmanov [1], Svetlana Solovieva [2] and Igor Antipin [1]

[1] Alexander Butlerov Institute of Chemistry, Kazan Federal University, 18 Kremlevskaya street, 420008 Kazan, Russia; mir_din@mail.ru (D.M.); kleo-w@mail.ru (F.G.); chem.khalifa21@yahoo.com (M.A.M.K.); avaliyahmetova@mail.ru (A.G.); nougmanoff@hotmail.com (R.N.); iantipin54@yandex.ru (I.A.)

[2] A.E.Arbuzov Institute of Organic & Physical Chemistry, 8 Arbuzov street, 420088 Kazan, Russia; skleshni@iopc.ru (S.K.); evgersol@yandex.ru (S.S.)

* Correspondence: ultrav@bk.ru; Tel.: (+7-843-2337344)

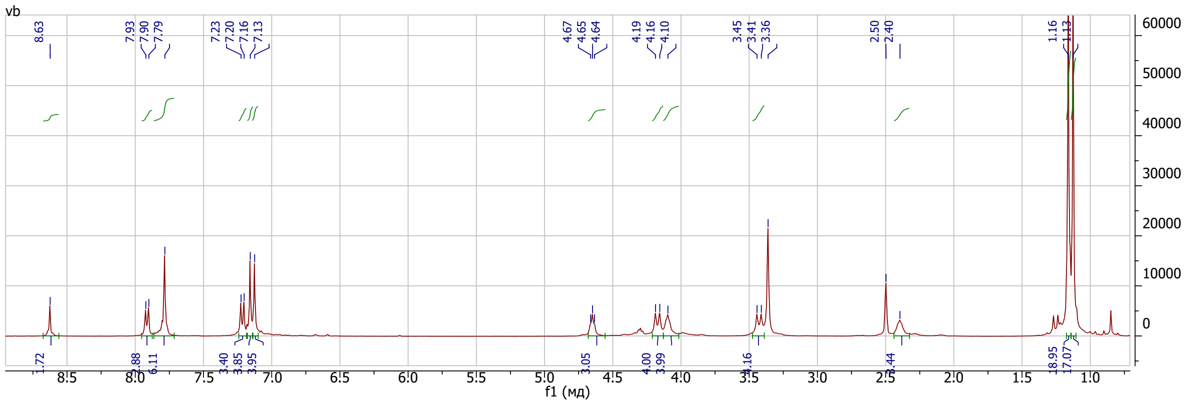
vb
8.63
7.93
7.90
7.79
7.23
7.20
7.16
7.13
4.67
4.65
4.64
4.19
4.16
4.10
3.45
3.41
3.36
2.50
2.40
1.16
1.13
1.72
2.88
6.11
3.40
3.85
3.95
3.05
4.00
3.99
4.16
2.44
18.95
17.07
60000
50000
40000
30000
20000
10000
0
8.5
8.0
7.5
7.0
6.5
6.0
5.5
5.0
4.5
4.0
3.5
3.0
2.5
2.0
1.5
1.0
f1 (мд)

a)

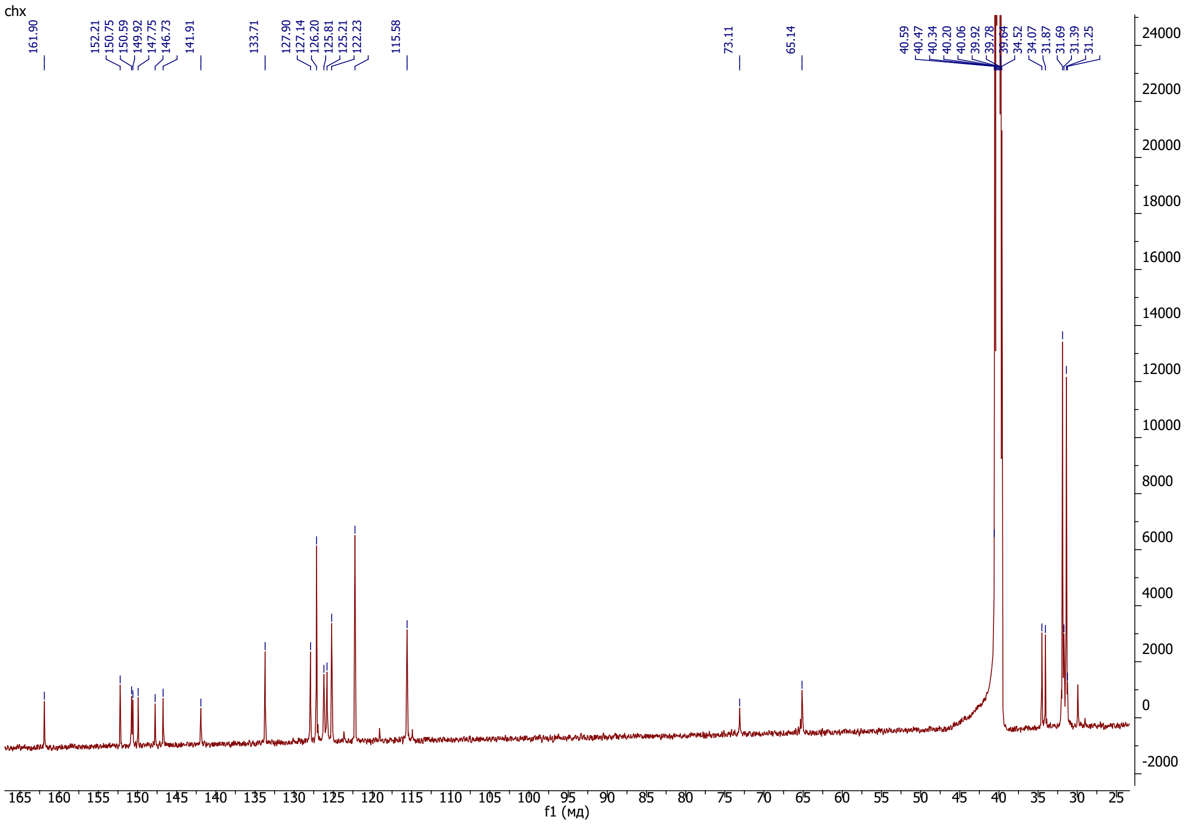
chx
161.90
152.21
150.75
150.59
149.92
147.75
146.73
141.91
133.71
127.90
127.14
126.20
125.81
125.21
122.23
115.58
73.11
65.14
40.59
40.47
40.34
40.20
40.06
39.92
39.78
39.64
34.52
34.07
31.87
31.69
31.39
31.25
24000
22000
20000
18000
16000
14000
12000
10000
8000
6000
4000
2000
0
-2000
165
160
155
150
145
140
135
130
125
120
115
110
105
100
95
90
85
80
75
70
65
60
55
50
45
40
35
30
25
f1 (мд)

b)

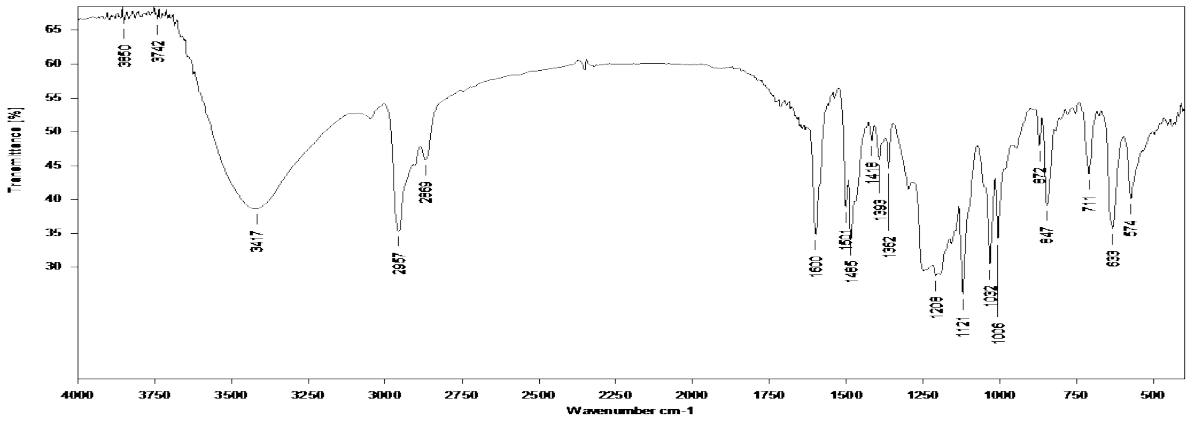
Transmittance [%]
65
60
55
50
45
40
35
30
3850
3742
3417
2957
2869
1600
1501
1485
1419
1393
1362
1208
1121
1032
1006
872
847
711
633
574
4000
3750
3500
3250
3000
2750
2500
2250
2000
1750
1500
1250
1000
750
500
Wavenumber cm-1

c)

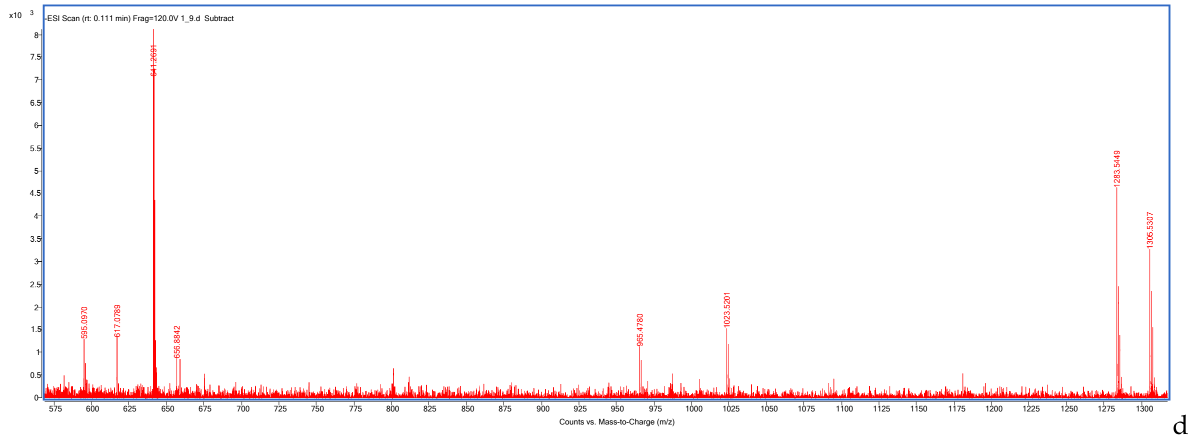

d)

Figure S1. NMR ^{1}H (a), ^{13}C (b), IR (c) and HRESI-MS (c) spectra of 25,27-bis(4-((4-sulfophenyl)diazenyl)phenyl)-propoxy)-26,28-dihydroxy-5,11,17,23-tetra(tert-butyl)calix[4]arene **4**.

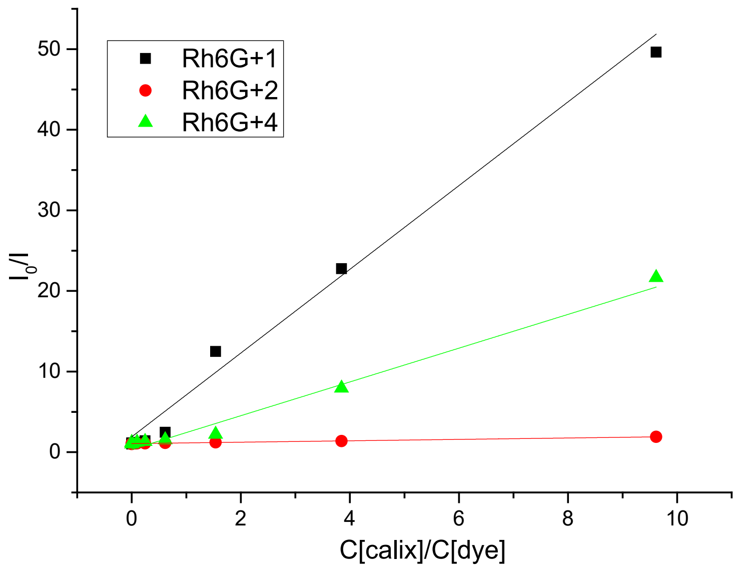

Figure S2. Stern Volmer plots for Rhodamine 6G with macrocycles **1**, **2**, **4**. C (dye) = 1 µM, PBS buffer (pH 7.4) at 37℃.

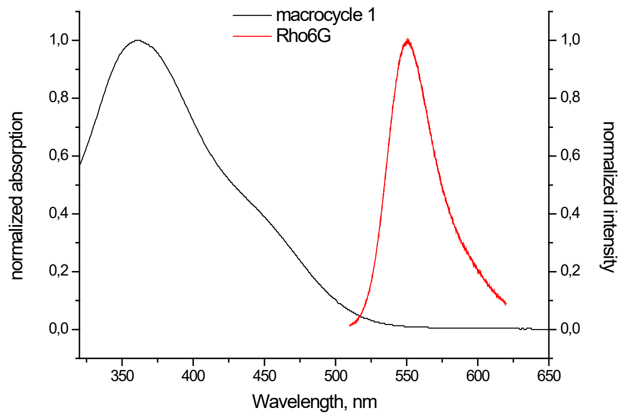

Figure S3. Normalized emission spectrum of Rho6G and absorption spectrum of **1** in PBS buffer (pH 7.4) at 37℃.

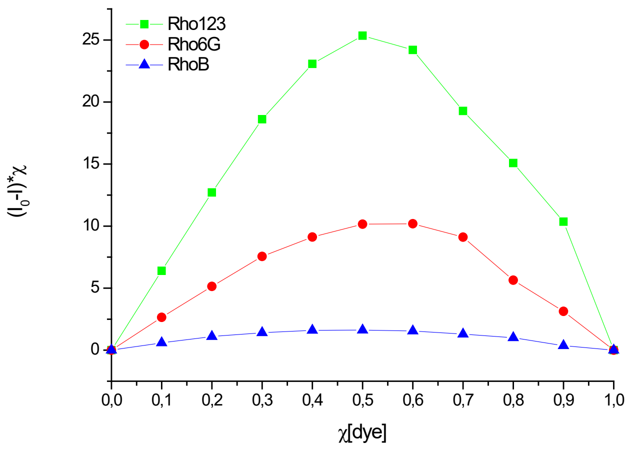

Figure S4. Job's plot for Rhodamine dyes with macrocycle **1**. C [total] = 2μM, PBS buffer (pH 7.4) at 37℃.

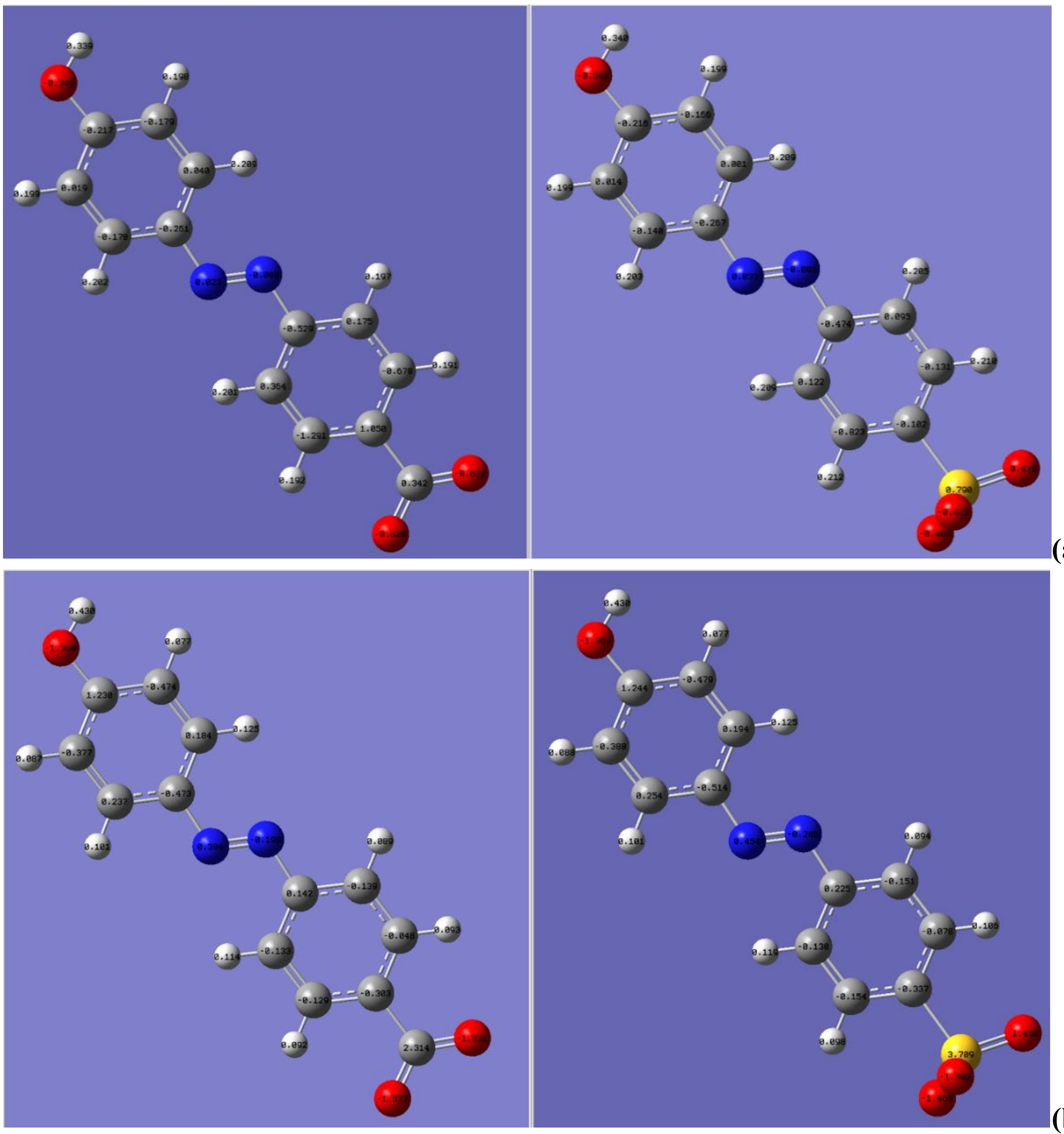

(a)

(b)

Figure S5. Mulliken and APT charges in model 4-((4-hydroxyphenyl)diazenyl)benzoate (left) and 4-((4-hydroxyphenyl)diazenyl)benzenesulfonate (right).

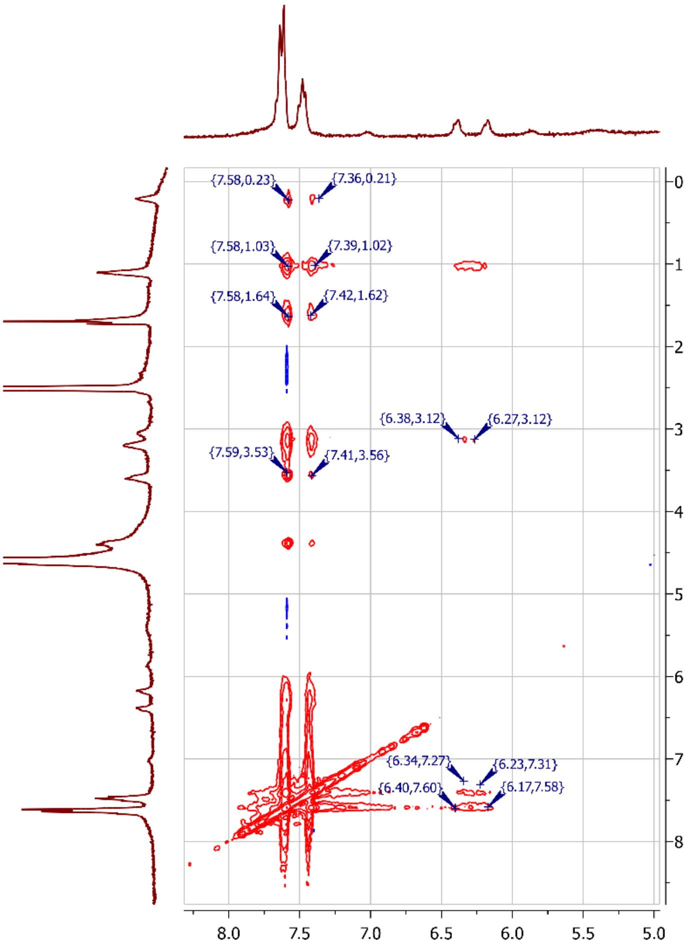

Figure S6. A fragment of 2D NOESY ^{1}H-^{1}H spectra of **1** with Rh6G, 0.5mM Rho6G and 1 mM of calixarene in DMSO-d6:D_2O (2:3) at 25 ℃, 10 mM PBS buffer (pH 7.4).

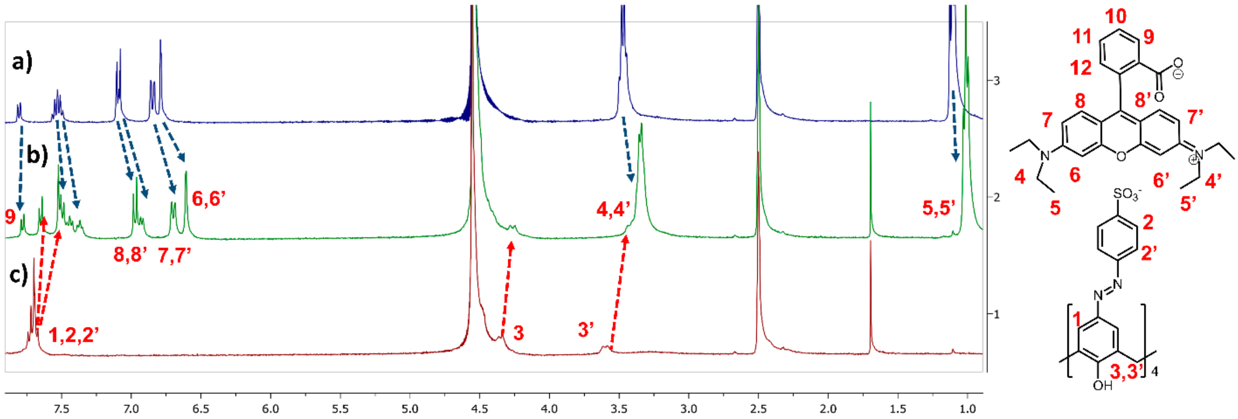

Figure S7. ^{1}H NMR spectra of 0.5mM RhoB (a), 1 mM calixarene **1** (c) and their (0.5:1) mixture (b) in DMSO-d6:D_2O (2:3) at 25 ℃, 10 mM PBS buffer (pH 7.4).

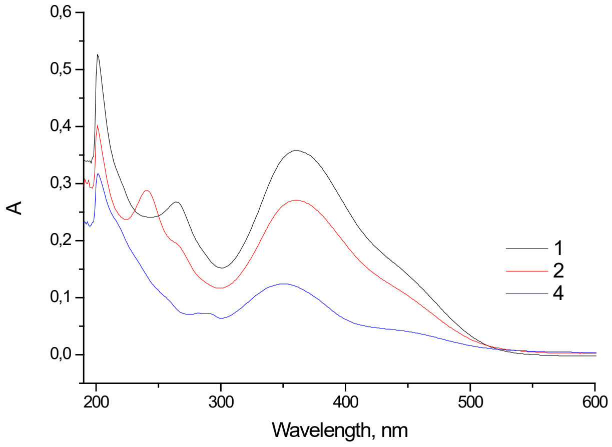

Figure S8. Absorption spectra of macrocycles **1**, **2**, **4** in PBS buffer.

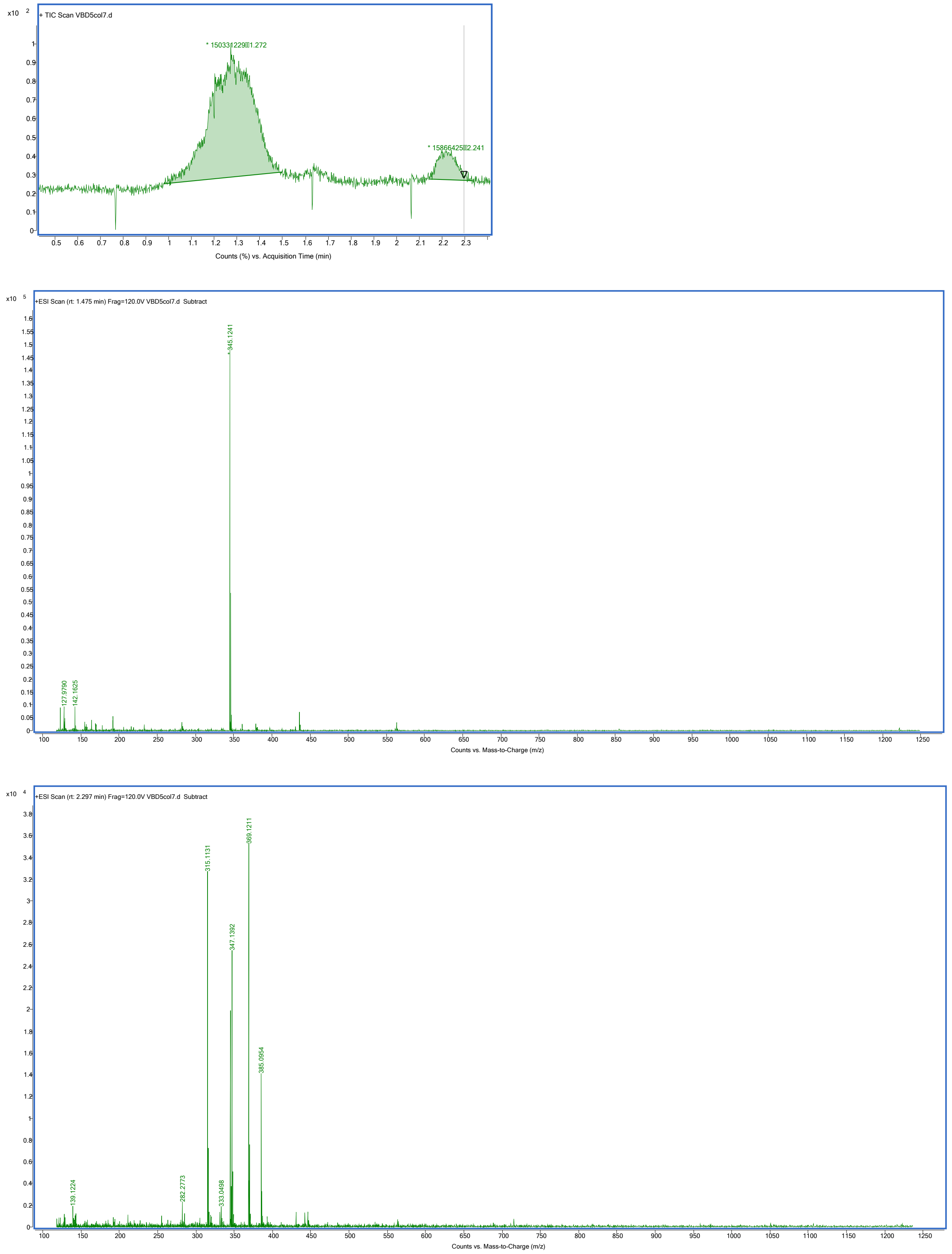

Figure S9. Chromatogram and HRESI-mass spectra of Rh123, treated with 100-fold excess of SDT.

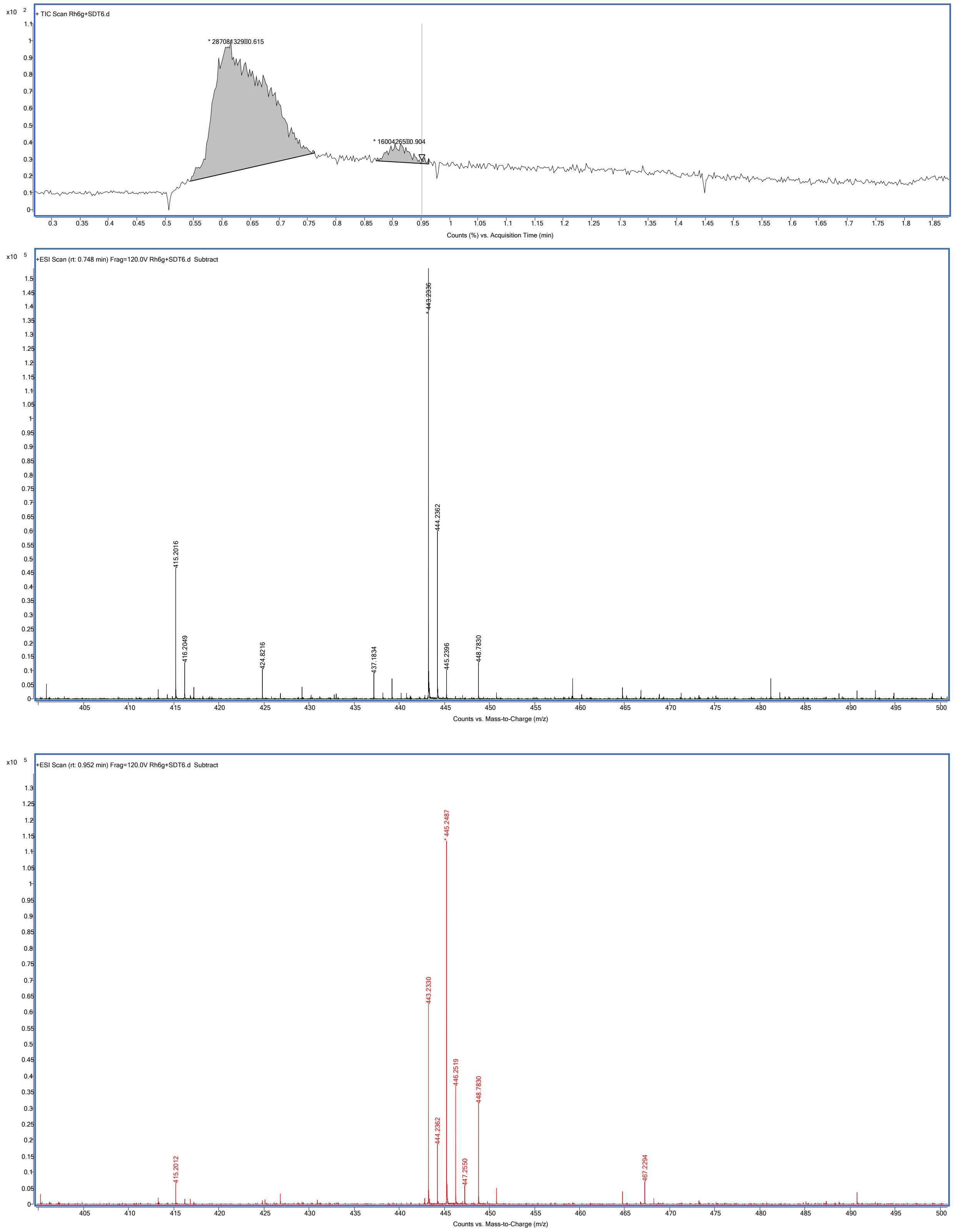

Figure S10. Chromatogram and HRESI-mass spectra of Rh6G, treated with 100-fold excess of SDT.

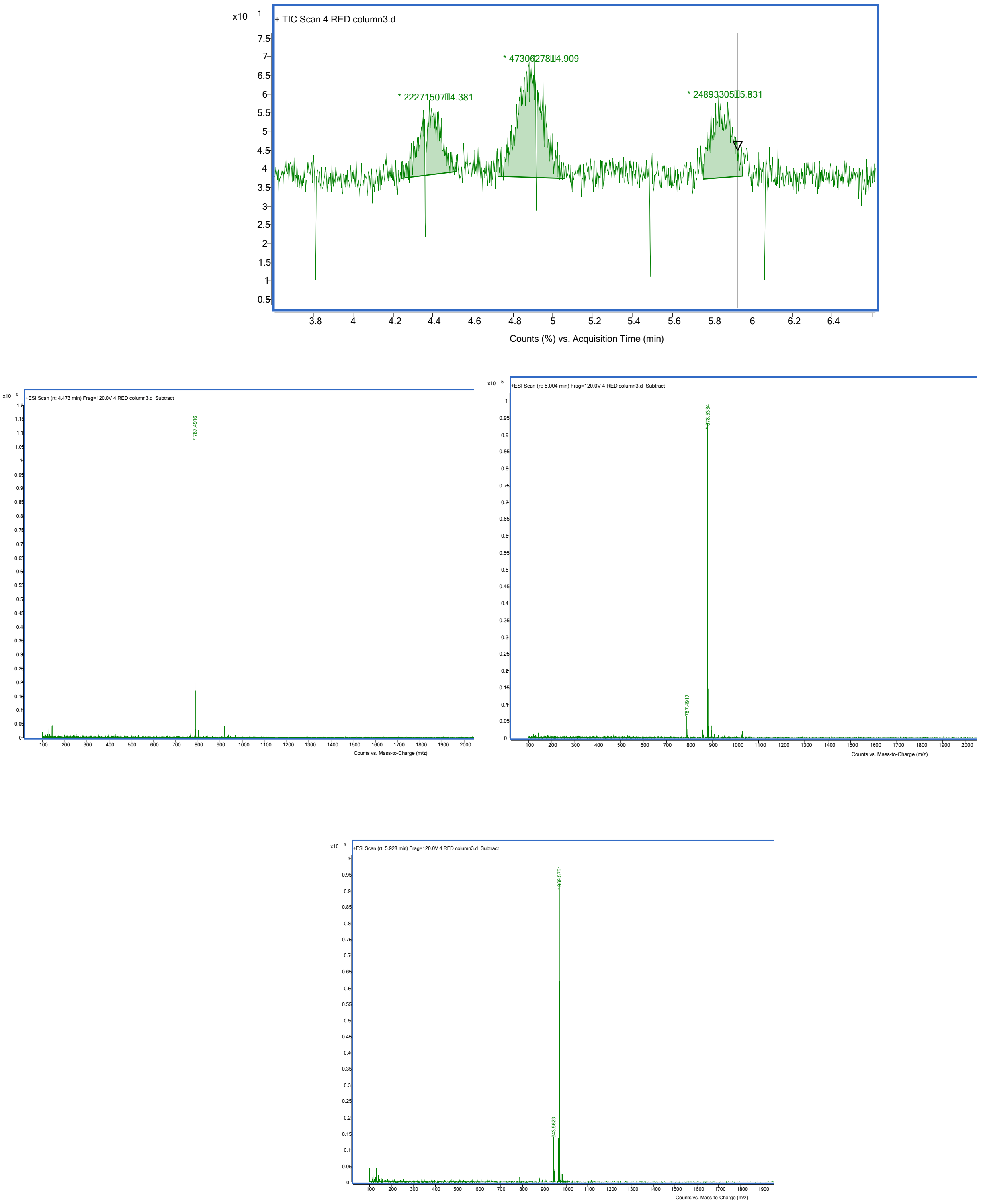

Figure S11. Chromatogram and HRESI-mass spectra of **4**, treated with 100-fold excess of SDT.
